# Causal relationship between particulate matter 2.5 (PM_2.5_), PM_2.5_ absorbance, and COVID-19 risk: A two-sample Mendelian randomisation study

**DOI:** 10.7189/jogh.13.06027

**Published:** 2023-07-14

**Authors:** Chenxi Liu, Jia Peng, Yubo Liu, Yi Peng, Yuanyuan Kuang, Yinzhuang Zhang, Qilin Ma

**Affiliations:** 1 Department of Cardiovascular Medicine, Xiangya Hospital, Central South University, Changsha, Hunan, China; 2 National Clinical Research Center for Geriatric Disorders, Xiangya Hospital, Changsha, Hunan, China; 3 Department of Rheumatology and Immunology (T.X.), Xiangya Hospital, Central South University, Changsha, Hunan, China; 4 Department of Cardiovascular Medicine, The First Hospital of Changsha, Changsha, Hunan, China

## Abstract

**Background:**

Several observational studies reported on the association between particulate matter ≤2.5μm (PM_2.5_) and its absorbance with coronavirus (COVID-19), but none use Mendelian randomisation (MR). To strengthen the knowledge on causality, we examined the association of PM_2.5_ and its absorbance with COVID-19 risk using MR.

**Methods:**

We selected genome-wide association study (GWAS) integration data from the UK Biobank and IEU Open GWAS Project for two-sample MR analysis. We used inverse variance weighted (IVW) and its multiple random effects and fixed effects alternatives to generally predict the association of PM_2.5_ and its absorbance with COVID-19, and six methods (MR Egger, weighted median, simple mode, weighted mode, maximum-likelihood and MR-PRESSO) as complementary analyses.

**Results:**

MR results suggested that PM_2.5_ absorbance was associated with COVID-19 infection (odds ratio (OR) = 2.64; 95% confidence interval (CI) = 1.32-5.27, *P* = 0.006), hospitalisation (OR = 3.52; 95% CI = 1.05-11.75, *P* = 0.041) and severe respiratory symptoms (OR = 28.74; 95% CI = 4.00-206.32, *P* = 0.001) in IVW methods. We observed no association between PM_2.5_ and COVID-19.

**Conclusions:**

We found a potential causal association of PM_2.5_ absorbance with COVID-19 infection, hospitalisation, and severe respiratory symptoms using MR analysis. Prevention and control of air pollution could help delay and halt the negative progression of COVID-19.

Coronavirus disease 2019 (COVID-19), a globally prevalent infectious disease caused by the severe acute respiratory syndrome coronavirus-2 (SARS-CoV-2) [1], leads to severe respiratory symptoms [2] and pathological lung changes like ground-glass opacities, signs of reticulation (including course fibrous bands, either with or without obvious parenchymal distortion), bronchiectasis, pulmonary fibrosis [3-5], and complications of multiple body systems [6,7], which is seriously harmful to human health.

Evidence suggests that exposure to air pollution is related to susceptibility to SARS-CoV-2 infection and COVID-19 severity [8]. Particulate matter ≤2.5μm (PM_2.5_) is a significant component of air pollutants and extremely detrimental to human health because of their small size [9], due to which it reduces lung immune response and antibacterial activity and increases viral load. Recent studies have shown that PM_2.5_ is possibly related to known COVID-19 symptoms and mortality [10-12]. PM_2.5_ increase of 1μg*m^-3^ can lead to at least an 11% increase in COVID-19 mortality in the USA [13]. However, evidence of these associations comes from observational studies rather than randomised controlled trials, preventing conclusion regarding association due to possible confounding.

As no studies have reported or discussed the use of the genetic instruments to predict the correlation between PM_2.5_ and COVID-19 risk, we hypothesised that there may exist a causal relationship between PM_2.5_ and COVID-19 risk. We conducted a two-sample Mendelian randomisation (MR) study to investigate the association between PM_2.5_, PM_2.5_ absorbance (a proxy of elemental carbon) [14], and COVID-19 risk.

## METHODS

### Study design

The MR design, which minimises the impact of environmental and other confounding factors, is based on genetic variation as instrument variables of exposure factors, and infers the causal relationship between exposure factors and outcome variables. This method's random allocation of alleles is similar to that of randomized controlled trials [15,16]. Additionally, it can increase the directivity of causality and diminish reverse causation, because the process cannot change the genetic variation of exposure factors.

**Figure 1:**
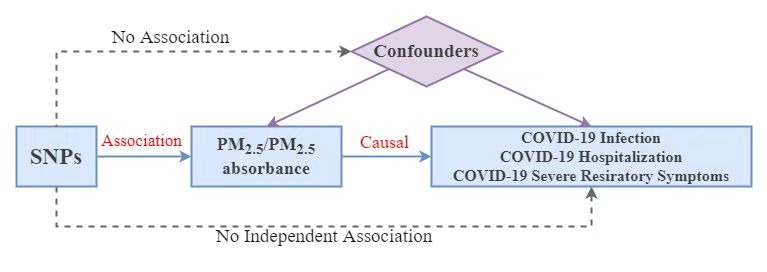
Study flowchart.

Using genetic variants as instrumental variables, MR analysis consists of three essential assumptions (**Figure 1**). The first assumption is that the genetic variants proposed as instrumental variables should be robustly associated with exposure, the second indicates that the used genetic variants should not be associated with any confounder factors, and the third is that the selected genetic variants should affect the risk of the outcome only through risk factors.

### Screen of genetic instrument

We obtained single-nucleotide polymorphisms (SNPs) as instrumental variables associated with PM_2.5_ and PM_2.5_ absorbance from genome-wide association study (GWAS) data sets of IEU’s analysis of the UK Biobank, containing 423 796 individuals of European ancestry. These data sets can be searched in the IEU Open GWAS Project (GWAS ID: ukb-b-11312, and ukb-b-10817) [17]. To select the most powerful instrumental variables, we grouped the data set screen standards (*P* < 5 × 10^−8^, r^2^<0.01, and clump distance >10 000kb) [18] to eliminate the linkage disequilibrium for excluding potential horizontal pleiotropy and insignificant SNPs. We then selected eight independent SNPs associated with PM_2.5_ and five independent SNPs associated with PM_2.5_ absorbance.

### COVID-19 data source

We obtained summary data on the association of COVID-19 cases from the European Bioinformatics Institute (EBI) database of complete GWAS summary data, searchable through the IEU Open GWAS Project [17]. We chose three data sets to investigate different COVID-19 situations, including COVID-19 (1 644 784 controls, 38 984 cases), recent COVID-19 hospitalisations (1 549 095 controls, 8316 cases) and confirmed COVID-19 cases with severe respiratory symptoms (1 383 241 controls, 5101 cases) [19]. We excluded all individuals who had withdrawn consent from either data source.

### Statistical analysis

We performed the inverse variance weighted (IVW) method and its multiple random effects and fixed effects alternatives to estimate the association for genetically predicted PM_2.5_ and PM_2.5_ (analysed SNPs >3) [18,20]. Additionally, MR-Egger [21], weighted median [22], simple mode, weighted mode, maximum-likelihood [23], and MR-PRESSO [24] were used as complementary analysis to IVW.

We conducted the two-sample MR analysis between two groups of selected SNPs and the above three groups of COVID-19, respectively. The total odds ratio (OR) was the effect of PM_2.5_ and PM_2.5_ absorbance on COVID-19 separately. We interpreted a *P*-value <0.05 as statistically significant. Sensitivity analyses consisted of three parts and several methods. First, we assessed the heterogeneity using the value of Cochran’s Q test, with a *P*-value <0.05 suggesting that heterogeneity existed [25], but the IVW method results with the multiplicative random-effects model were still reliable in this situation. Second, we assessed horizontal pleiotropy [26] to avoid the second and third assumption, calculating it using MR-Egger intercept [21]. If the *P*-value of MR-Egger intercept was <0.05, we considered the effect of SNPs associated with exposure factors on outcomes as unreliable. Third, we performed the leave-one-out analysis, excluding each SNP one by one to determine whether a single SNP significantly changed the results [27]. Using the IVW method, we could calculate the “all” numerical value, and considered the results reliable if “all”>0. Additionally, the MR-PRESSO method can recognise outliers (SNPs) and provide a causal estimate after corresponding outliers are removed and the forest plot reflected the correlation of exposure factors with outcomes in each SNP. To avoid weak instrumental bias, we used the *F* statistic to measure the strength of instrument variables. If *F* was >10, we considered the outcome to be unaffected by weak instruments [28,29]. We performed all analyses in R (Version 4.1.2) using the “TwoSampleMR” [30] and “MR-PRESSO” packages.

## RESULTS

### Information of selected SNPs

**Figure 2:**
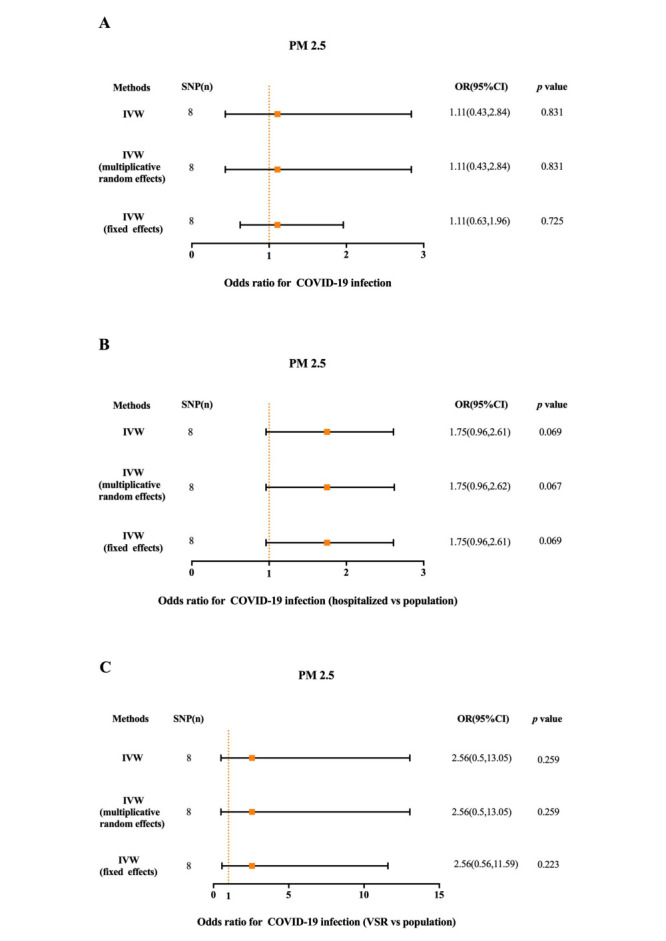
The MR results of PM_2.5_ on the status of COVID-19. **Panel A.** The association of PM_2.5_ with COVID-19 infection. **Panel B.** The association of PM_2.5_ with COVID-19 hospitalization. **Panel C.** The association of PM_2.5_ with COVID-19 with VSR. SNP – single-nucleotide polymorphisms, OR – odds ratio, IVW – the inverse variance weighted method, VSR – very severe respiratory symptoms.

**Figure 3:**
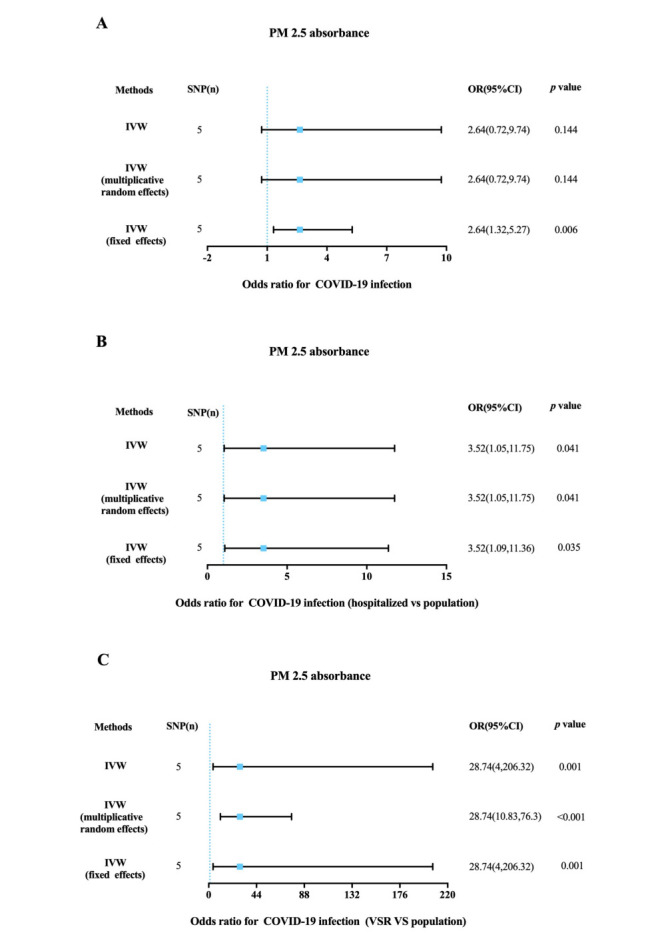
The MR results of PM_2.5_ absorbance on the status of COVID-19. **Panel A.** The association of PM_2.5_ absorbance with COVID-19 infection. **Panel B.** The association of PM_2.5_ absorbance with COVID-19 hospitalization. **Panel C.** The association of PM_2.5_ absorbance with COVID-19 with VSR. SNP – single-nucleotide polymorphisms, OR – odds ratio, IVW – the inverse variance weighted metho, VSR – very severe respiratory symptoms.

The *F* statistic was greater than 10 for all the instrument variables associated with PM_2.5_ and PM_2.5_ absorbance in UK Biobank study (**Table 1** and **Table 2**). The MR analysis estimated the risk of PM_2.5_ and PM_2.5_ absorbance on COVID-19 of status (**Figure 2**, **Figure 3**, and Figure S1, Table S1 and S2 in the **Online Supplementary Document**). Through MR-PRESSO, outlier SNPs have been eliminated and statistics have been corrected.

**Table 1:** Selected genetic instruments of PM_2.5_

SNP	Chr	Beta	SE	*P*-value	F	Related genes
rs114708313	6	0.025	0.004	4.20E-08	30.076	HCG27
rs12203592	6	0.022	0.003	6.20E-17	69.918	IRF4
rs1372504	5	0.012	0.002	3.10E-08	30.674	NONE
rs1537371	9	0.012	0.002	8.50E-09	33.149	CDKN2B-AS1
rs6749467	2	-0.012	0.002	1.40E-08	32.228	FAM150B
rs72642437	18	0.113	0.019	3.10E-09	35.119	ZBTB7C
rs77205736	8	0.014	0.002	2.10E-08	31.399	MSRA
rs77255816	6	0.031	0.006	4.20E-08	30.041	CDKAL1

SNPs – single-nucleotide polymorphisms, Chr – chromosome, SE – standard error, F – F statistics, HCG27 – HLA complex group 27, IRF4 – interferon regulatory factor 4, NONE – no related gene, CDKN2B-AS1 – CDKN2B antisense RNA 1, FAM150B – ALK and LTK ligand 2, ZBTB7C – zinc finger and BTB domain containing 7C, MSRA – methionine sulfoxide reductase A, CDKAL1 – CDK5 regulatory subunit associated protein 1 like 1

**Table 2:** Selected genetic instruments of PM_2.5_ absorbance

SNP	Chr	Beta	SE	*P*-value	F	Related genes
rs12203592	6	0.017	0.003	1.20E-10	41.539	IRF4
rs4915350	1	0.046	0.008	5.70E-09	33.933	LINC02789
rs59727727	6	0.018	0.003	2.80E-08	30.823	MICA
rs77205736	8	0.013	0.002	4.50E-08	29.911	MSRA
rs79475047	6	0.040	0.007	1.60E-09	36.427	CDKAL1

SNPs – single-nucleotide polymorphisms, Chr – chromosome, SE – standard error, F – F statistics; IRF4 – interferon regulatory factor 4, LINC02789 – long intergenic non-protein coding RNA 2789, MICA – MHC class I polypeptide-related sequence A, MSRA – methionine sulfoxide reductase A, CDKAL1 – CDK5 regulatory subunit associated protein 1 like 1

### MR analysis of PM_2.5_ on the status of COVID-19

Using screened instrument variables, we found no association between PM_2.5_ and COVID-19 in MR. In the IVW MR analysis, genetically predicted PM_2.5_ was not significantly associated with COVID-19 infection (OR = 1.11; 95% confidence interval (CI) = 0.43-2.84, *P* = 0.831)), hospitalisation (OR = 1.75; 95% CI = 0.96-2.61, *P* = 0.068) and severe respiratory symptoms (OR = 2.56; 95% CI = 0.50-13.05, *P* = 0.259) vs population (**Figure 2**). In the sensitivity analysis (Table S3 in the **Online Supplementary Document**), we estimated that there were confounding factors and horizontal pleiotropy between instrumental variables of PM_2.5_ and COVID-19 infection (Cochran’s Q: *P* = 0.012, MR-Egger: *P* = 0.018), which indicated that the results of two-sample MR in this group were unreliable. Although there was not significance in the IVW method, instrument variables in weight median method (Figure S1 and Table S1 in the **Online Supplementary Document**) showed significant statistical differences between PM_2.5_ and COVID-19 hospitalisation (OR = 2.06; 95% CI = 1.04-2.84, *P* = 0.038).

### MR analysis of PM_2.5_ absorbance on the status of COVID-19

In the IVW MR analysis, genetically predicted PM_2.5_ absorbance was associated with COVID-19 hospitalisation (OR = 3.52; 95% CI = 1.05-11.75, *P* = 0.041) and severe respiratory symptoms (OR = 28.74; 95% CI = 4.00-206.32, *P* = 0.001) (**Figure 3**). Although the association of PM_2.5_ absorbance with COVID-19 infection was not significantly statistical in the IVW method (OR = 2.64; 95% CI = 0.72-9.74, *P* = 0.144), there was differences in the methods of IVW (fixed effects) (OR = 2.64; 95% CI = 1.32-5.27, *P* = 0.006), weighted median (OR = 3.93; 95% CI = 1.28-12.08, *P* = 0.017) and maximum likelihood (OR = 2.85; 95% CI = 1.37-5.95, *P* = 0.005) (Table S2 in the **Online Supplementary Document**). In the sensitivity analysis (Table S3 in the **Online Supplementary Document**), the heterogeneity test detected heterogeneity between instrumental variables of PM_2.5_ absorbance and COVID-19 infection, but no horizontal pleiotropy was observed between genetic instruments and all outcomes, which did not affect the reliability of the results.

## DISCUSSION

We obtained and filtered relevant genome-wide data from GWAS as genetic instruments to explore the causal association of PM_2.5_ and PM_2.5_ absorbance with infection, hospitalisation, or severe respiratory symptoms of COVID-19. We found that PM_2.5_ absorbance may be associated with increased risk of COVID-19 hospitalisation and severe respiratory symptoms. We also found PM_2.5_ absorbance was a risk factor for COVID-19 prevalence in weight median and maximum likelihood method. However, there was no significant relationship between exposure to PM_2.5_ and COVID-19 infection, hospitalisation, or severe respiratory symptoms.

PM_2.5_ absorbance, as a proxy and indicator of element carbon reflecting the concentration of carbonaceous components in PM_2.5_, may increase the risk of COVID-19 infection and deterioration by reducing the resistance to infection by negatively influencing lung structure and long-term function [31,32]. Consistently, PM_2.5_ absorbance mainly determined by exposure to environmental tobacco smoke indoors [33] was most likely associated with worse progression and adverse outcomes of COVID-19 [34]. Studies have shown that PM_2.5_ absorbance was significantly correlated with brain malignancy, incident hypertension and metabolic syndrome, which could exacerbate the severity of COVID-19 by affecting body physiologic function, including endothelial dysfunction and abnormal lipids metabolism [35-37].

Previous studies provided a pathogenetic explanation for this association. In a large cohort study, PM_2.5_ absorbance was found to be possibly associated with the increase of gamma glutamyl transferase (GGT) [38], which was linked to the occurrence of accumulating inflammation response and applications in COVID-19 [39,40]. Furthermore, a mice model study indicated that PM_2.5_ carbonaceous components such as elemental carbon drive an acute cardiovascular response by increasing blood pressure and heart rate, which may increase cardiovascular burden and potential risk for complications in COVID-19 patients [41]. The carbonaceous particles in PM_2.5_ may act as vehicles for strong acids like H_2_SO_4_ and cause damage to alveolar epithelium with inhalation of PM_2.5_, decreasing resistance to infection and pulmonary dysfunction [42]. Additionally, inhalation of high levels of spherical carbonaceous nanoparticles may induce inflammatory response and reactivates latent virus [43,44]. Through the IVW methods, we found PM_2.5_ absorbance was a likely risk factor of COVID-19 hospitalisation and severe respiratory symptoms. Meanwhile, PM_2.5_ absorbance may also increase the risk of COVID-19 infection estimated in the fixed effects IVW, weighted median and maximum likelihood method. Based on multiple statistical models, PM_2.5_ absorbance was identified to be possibly associated with increased risk of COVID-19 prevalence and negative progression.

Some studies have suggested PM_2.5_ absorbance was possibly a risk factor for COVID-19 cases, increasing patients’ symptoms and mortality [45-50]. A prospective cohort study showed that PM_2.5_ was significantly associated with COVID-19 hospitalisations and accesses to intensive care units [51], while another study reported that pollution stemming from PM_2.5_ caused poor prognosis of COVID-19 patients [52]. However, confounding factors are often present, and we also found no adequate evidence for the association of PM_2.5_ with COVID-19, even though the result of weight median method showed significant statistical differences between PM_2.5_ and COVID-19 with hospitalisation.

Components of PM_2.5_ may provide a reasonable explanation for the difference between the associations of PM_2.5_ and PM_2.5_ absorbance with COVID-19 risk. These components are intricate and mainly contain carbonaceous aerosol (including elemental carbon and organic carbon particles like polycyclic aromatic hydrocarbon), crustal components, trace elements, and heavy metals, which trigger various pathogenic mechanisms [53-55]. When analysing PM_2.5_ as a whole factor, we found no association with COVID-19 in the MR, but discovered that PM_2.5_ absorbance (as a proxy of elemental carbon that accounts for 50% proportion of PM_2.5_) was possibly correlated with COVID-19 risk, suggesting that carbonaceous components in PM_2.5_ may be independently associated with COVID-19 risk, but that other compositions probably weaken or inversely interfere in this association with PM_2.5_ [56]. Considering GWAS data of PM_2.5_ only originated from the European population, further genetic studies are necessary to confirm our findings in other races and circumstances.

Although this study has several strengths, it also has some limitations. First, GWAS data only came from European populations, and we could not assess the effect of age and sex on the observed association in summary level data. We thus lack genetic data for different regions, races, and environments, so we cannot generalise our findings to other populations or contexts. Second, we detected heterogeneity and horizontal pleiotropy, and had a limited number of SNPs in our analysis, making the correlation between PM_2.5_ and COVID-19 infection unreliable. The source of this heterogeneity might have been the different detection methods used for obtaining corresponding data. In some cases, we can still trust the results with heterogeneity in IVW methods [57], but may need to rescreen the tool variables or collect new GWAS data to exclude the impact of horizontal pleiotropy. Additionally, the accuracy of the statistical model may be affected by a high standard error due to the low number of genetic instruments, which indicates we need to constantly update relevant GWAS data. Third, since PM_2.5_ consists of multiple pollutants, and as there is no clear PM_2.5_ component in the summary level data, we cannot conduct further subgroup analyses to determine its effect on COVID-19. Finally, several, often more harmful variations of SARS-CoV-2 (eg, Omicron) emerged in different geographical areas since 2021 [58,59]. Our data was only collected in 2020 and do not contain data on the adverse effects of new viral strain on a person’s body or genes, meaning updated data could further improve the relevance of our study. Meanwhile, indicators of environmental pollution such as PM_2.5_ concentration, should be further analysed through linear or nonlinear MR studies to make our conclusion more reliable.

## CONCLUSIONS

We found a potentially casual association of PM_2.5_ absorbance with COVID-19 infection, hospitalisation, and severe respiratory symptoms using MR analysis. Prevention and control of air pollution may help delay and block the negative progression of COVID-19.

## Additional material


Online Supplementary Document
