# Supplementary material for: Causal relationship between particulate matter 2.5 (PM2.5), PM2.5 absorbance, and COVID-19 risk: A two-sample Mendelian randomisation study
Source: J Glob Health. 2023 Jul 14;13:06027. doi: 10.7189/jogh.13.06027 (PMC10346132; doi:10.7189/jogh.13.06027)
Supplement: Online Supplementary Document [file jogh-13-06027-s001.pdf]

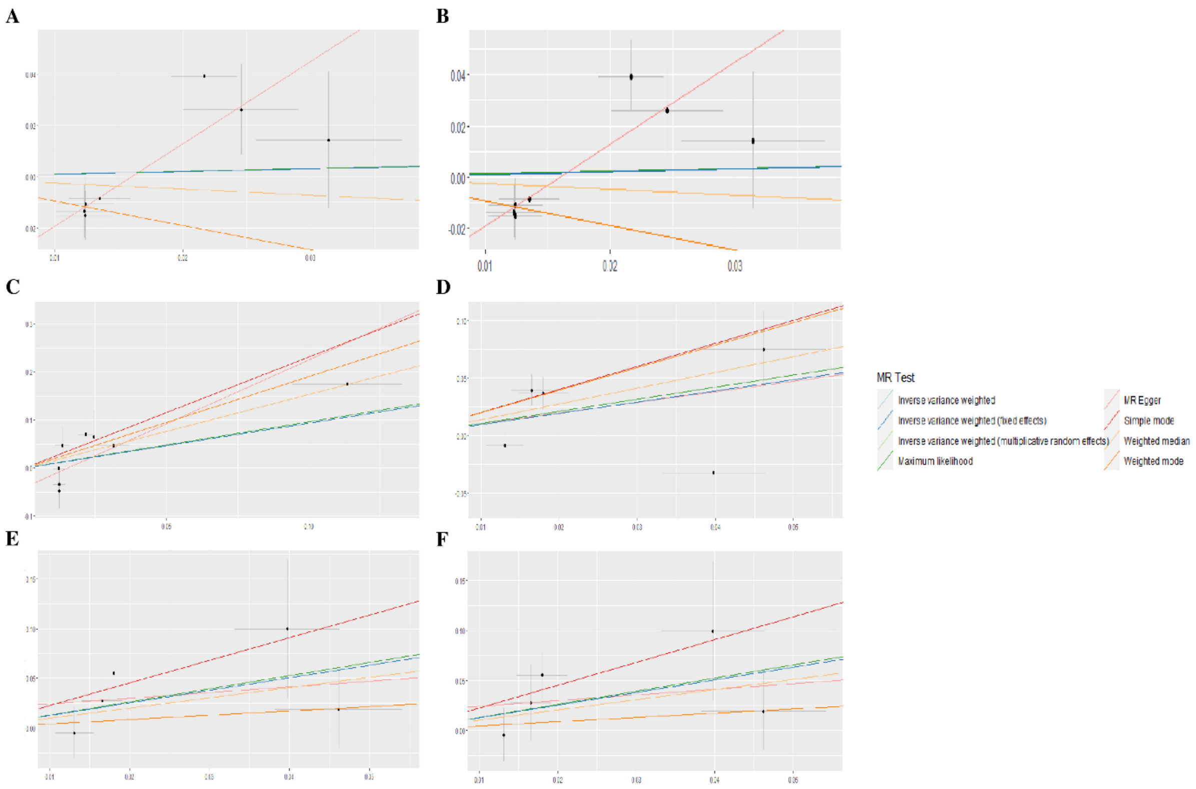

Figure S1. The results of Different MR analysis models on the association of PM2.5 and PM2.5 absorbance. **Panel A.** The association of $PM_{2.5}$ with COVID-19 infection; **Panel B.** The association of $PM_{2.5}$ with COVID-19 hospitalization; **Panel C.** The association of $PM_{2.5}$ with COVID-19 with VSR; **Panel D.** The association of $PM_{2.5}$ absorbance with COVID-19 infection, **Panel E.** The association of $PM_{2.5}$ absorbance with COVID-19 hospitalization, **Panel F.** The association of $PM_{2.5}$ absorbance with COVID-19 with VSR. VSR – very severe respiratory symptoms.

Table S1. The MR results of $PM_{2.5}$ with COVID-19

**Outcome/Models**	**OR (95%CI)**	***P*-val**
**COVID-19 infection**		
MR Egger	24.82 (3.93-156.83)	0.019
Weighted median	0.79 (0.33-1.88)	0.590
IVW	1.11 (0.43-2.84)	0.831
Simple mode	0.39 (0.08-1.79)	0.269
Weighted mode	0.39 (0.05-3.18)	0.410
Maximum likelihood	1.12 (0.62-2.01)	0.715
IVW (multiplicative random effects)	1.11 (0.43-2.84)	0.831
IVW (fixed effects)	1.11 (0.63-1.96)	0.725
MR-PRESSO	0.71 (0.33-1.56)	0.436
**COVID-19 hospitalization**		
MR Egger	2.72 (1.22-6.03)	0.037
Weighted median	2.06 (1.04-4.08)	0.038
IVW	1.75 (0.96-3.19)	0.069
Simple mode	2.15 (0.34-13.50)	0.446
Weighted mode	2.22 (1.10-4.49)	0.038
Maximum likelihood	1.74 (0.96-3.17)	0.069
IVW (multiplicative random effects)	1.75 (0.96-3.17)	0.067
IVW (fixed effects)	1.75 (0.96-3.19)	0.069
MR-PRESSO	1.75 (0.96-3.17)	0.116

**COVID-19 with VSR**		
MR Egger	14.16 (0.98-203.16)	0.100
Weighted median	4.64 (0.65-32.91)	0.125
IVW	2.56 (0.50-13.05)	0.259
Simple mode	10.12 (0.49-210.70)	0.179
Weighted mode	6.69 (0.51-88.52)	0.192
Maximum likelihood	2.61 (0.57-12.06)	0.219
IVW (multiplicative random effects)	2.56 (0.501-13.05)	0.259
IVW (fixed effects)	2.56 (0.56-11.59)	0.223
MR-PRESSO	2.56 (0.50-13.05)	0.296

P-val – *p*-value; OR – odds ratio; CI – confidence interval; IVW – inverse variance weighted; VSR – very severe respiratory symptoms.

Table S2. The MR results of $PM_{2.5}$ absorbance with COVID-19

Outcome/Models	OR (95%CI)	*P*-val
**COVID-19 infection**		
MR Egger	2.52 (0.07-89.63)	0.647
Weighted median	3.93 (1.28-12.08)	0.017
IVW	2.64 (0.72-9.74)	0.144
Simple mode	7.43 (1.51-36.57)	0.069
Weighted mode	7.10 (1.40-36.00)	0.077
Maximum likelihood	2.85 (1.37-5.95)	0.005
IVW (multiplicative random effects)	2.64 (0.72-9.74)	0.144
IVW (fixed effects)	2.64 (1.32-5.27)	0.006
MR-PRESSO	NA	NA
**COVID-19 hospitalization**		
MR Egger	1.75 (0.10-29.73)	0.723
Weighted median	2.77 (0.62-12.47)	0.184
IVW	3.52 (1.05-11.75)	0.041
Simple mode	9.66 (0.77-102.51)	0.153
Weighted mode	1.54 (0.32-7.45)	0.620
Maximum likelihood	3.73 (1.07-13.00)	0.039
IVW (multiplicative random effects)	3.52 (1.05-11.75)	0.041
IVW (fixed effects)	3.52 (1.09-11.36)	0.035
MR-PRESSO	NA	NA

**COVID-19 with VSR**		
MR Egger	8.65 (0.10-778.56)	0.417
Weighted median	48.51 (4.35-541.15)	0.002
IVW	28.74 (4.00-206.32)	0.001
Simple mode	62.38 (2.43-1598.16)	0.067
Weighted mode	60.27 (2.67-1358.76)	0.061
Maximum likelihood	29.91 (3.76-237.91)	0.001
IVW (multiplicative random effects)	28.74 (10.83-76.30)	< 0.001
IVW (fixed effects)	28.74 (4.00-206.31)	0.001
MR-PRESSO	NA	NA

P-val – *p*-value; OR – odds ratio; CI – confidence interval; IVW – inverse variance weighted; VSR – very severe respiratory symptoms; NA – not applicable.

Table S3. Heterogeneity and pleiotropy assessment

Exposure/Outcome	Cochran's Q	*P* for Cochran's Q	Egger intercept	*P* for intercept
**COVID-19 infection**				
$PM_{2.5}$	4.233	0.516	-0.051	0.018
$PM_{2.5}$ absorbance	14.259	0.003	0.001	0.978
**COVID-19 hospitalization**				
$PM_{2.5}$	3.140	0.678	-0.023	0.157
$PM_{2.5}$ absorbance	3.853	0.278	0.019	0.623
**COVID-19 with VSR**				
$PM_{2.5}$	5.810	0.445	-0.040	0.177
$PM_{2.5}$ absorbance	0.643	0.887	0.029	0.601

P – *p* value; VSR – very severe respiratory symptoms.
